# Prevalence of Enteropathogenic and Shiga Toxin-producing *Escherichia coli* among Children with and without Diarrhoea in Iran

**Published:** 2007-03

**Authors:** M. Yousef Alikhani, Akbar Mirsalehian, Bahram Fatollahzadeh, Mohammad R. Pourshafie, M. Mehdi Aslani

**Affiliations:** ^1^ Microbiology Department, Faculty of Medicine, Hamadan University of Medical Science, Hamadan, Iran; ^2^ Microbiology Department, Faculty of Medicine, Tehran University of Medical Science, Tehran, Iran; ^3^ Microbiology Department, Institute Pasteur of Iran, Tehran

**Keywords:** *Escherichia coli,* Enteropathogenic, *Escherichia coli,* Enterohaemorrhagic, Diarrhoea, Diarrhoea, Infantile, Colitis, Haemorrhagic, Diagnosis, Laboratory, Prevalence, Iran

## Abstract

The aim of the study was to determine the rates of detection of enteropathogenic *Escherichia coli* (EPEC) and Shiga toxin-producing *E. coli* (STEC) strains among children in two randomly-selected populations in Iran. In total, 1,292 randomly-selected faecal samples from children aged less than 10 years were screened for EPEC and STEC. Of the 1,292 cases participated in the study, 184 had diarrhoea, and 1,108 were healthy/asymptomatic children. The conventional culture method and slide agglutination with 12 different commercial EPEC antisera were used for the detection of EPEC. The colony sweep polymyxin-B extraction method, non-sorbitol fermentation (NSF) phenotype, and slide agglutination with O157:H7 antisera were used for the screening and detection of STEC. Of EPEC belonging to 11 different serogroups, O111 and O127 were most commonly found in 36.4% of the diarrhoeal cases and 7.2% of the asymptomatic children. A significant association (p<0.05) was found between isolation of EPEC and diarrhoea. 8.7% of the diarrhoeal cases and 2% of children without diarrhoea were infected with STEC, but none of the isolates belonged to the O157:H7 serotype. A significant association (p<0.05) was found between STEC and diarrhoeal cases. Based on these findings, it can be concluded that different EPEC serogroups may be agents of endemic infantile diarrhoea, and STEC strains are an important enteropathogen among young children.

## INTRODUCTION

Diarrhoea is one of the most common causes of morbidity and mortality among infants and young children in developing countries. The annual death toll in Asia, Africa, and America is estimated to be around 4.6–6 million (1, 2). In the past decades, diarrhoea was one of the major causes of infant deaths in Iran (3). However, the implementation of the World Health Organization-guided local programme of diarrhoeal disease control, including promotion of breastfeeding, oral rehydration therapy, and specific health education, has decreased the incidence of diarrhoeal diseases. At present, the figure approaches to 9.7 deaths per 1,000 births (www.who.int/child-adolescent-health/New_Publications/CHILD_HEALTH/CS/CS_paper _1.pdf)

*Escherichia coli,* a common cause of gastroenteritis, accounts for 30% of the total number of diarrhoeal pathogens in some regions (3, 4). Strains of *E. coli,* which cause diarrhoea in humans, can be divided into at least seven different categories based on distinct epidemiology, clinical syndromes, and virulence properties (5). The term enteropathogenic *E. coli* (EPEC) refers to *E. coli* strains of specific serotypes that epidemiologically incriminated as pathogens in outbreaks of diarrhoea. They are one of the main causes of severe infantile diarrhoea in many developing countries.

Serotyping is the classical method for the identification of strains of EPEC. Despite the development of new methods, such as DNA probe, polymerase chain reaction (PCR), and cell culture assays (6, 7), serotyping is still used as the main diagnostic tool. Twelve serogroups are known as the traditional serogroups of EPEC (8). Many workers have used serotyping as the method of investigating the prevalence of strains of EPEC among infants and young children with diarrhoea in different countries (2, 9–11). Of the EPEC serogroups identified in different areas, including Bangladesh (10), Saudi Arabia (11), and Venezuela (2), the prevalent serogroups in diarrhoeal cases were O114, O111, and O55 respectively.

Shiga toxin-producing *E. coli* (STEC) has been shown to be the most important group in developed countries because of numerous outbreaks and life-threatening complications, such as haemolytic-uraemic syndrome (HUS) and haemorrhagic colitis (HC) (5). However, self-limiting diarrhoea caused by STEC has frequently been encountered (12). Results of our studies showed that STEC is one of the diarrhoeal pathogens in Iran (13, 14). The higher rate of isolation in the summer season (4.9% in Ilam) (13) than in the spring season (0.7% in Mazandaran) encouraged us to find out the current situation of STEC-associated infection in the summer season in these areas.

The current study was conducted to determine the prevalence of STEC, besides EPEC, in the summer months. The aim of the study was to determine the rates of detection of EPEC and STEC strains in 1,292 children aged less than 10 years in two randomly-selected populations. We report here the epidemiological role of EPEC and STEC in children with and without diarrhoea.

## MATERIALS AND METHODS

### Study site and subjects

Two provinces in the north (Mazandaran province) and southwest (Ilam province) regions of Iran with populations of over four million and five hundred were selected respectively. In Mazandaran province, the climate is cooler and wetter, whereas Ilam province has a tropical climate with diarrhoea as a major public-health problem. Moreover, economic and cultural conditions are also different. A two-stage random-sampling method was employed for the collection of samples. The total populations of Ilam and Mazandaran provinces, according to population size, were divided into 400 and 500 blocks respectively. Fifty of these blocks (urban and rural) in every province were selected in the first stage. In the second stage, we chose randomly a neighbourhood in each block for sampling, and 8 and 15 families were then selected in Ilam and Mazandaran province respectively.

The study was conducted during the summer season of 2003, and 1,320 individuals aged less than 10 years—640 from Ilam province and 680 from Mazandaran province—were selected with a precision of 0.5% and confidence limit of 95%.

### Collection of faecal samples

A questionnaire was used for obtaining different clinical and personal data for each child. Diarrhoea was defined based on the number of defaecations per 24 hours and form of the stool. Three times or more watery or soft defaecations were considered diarrhoea. Swabs were inoculated with whole stool by families and were then transported to laboratory, using Cary and Blair's medium, within 12 hours after interview.

### Microbiological studies

The samples were cultured for enteric pathogens by conventional methods. *E. coli* O157:H7 was cultured on sorbitol-MacConkey agar (SMAC) (15). In brief, stool samples were cultured on *Salmonella-Shigella* (SS) agar, selenite broth for *Salmonella* and *Shigella,* on double plates of MacConkey agar (one at 37 ºC and the other at 25 ºC) for *Yersinia* spp. and on TCBS agar for *Vibrio* and *Aeromonas* spp. (16). Presumptive colony identification was carried out according to Edwards and Ewings (17). From SMAC at least five non-sorbitol-fermenting (NSF) colonies, if any, were stocked and serotyped with *E. coli* O157:H7 antisera.

### Detection of Shiga toxin-producing *E. coli*

Cytotoxic activity was tested on vero cell monolayer (15). The colony sweep polymyxin-B extraction method (18) was employed for extraction of Shiga toxin (stx) from MacConkey agar plates as described previously (13, 14). Strains of *E. coli*-producing Shiga toxin-1 (stx1) (19) and Shiga toxin-2-(stx2) (20) were used as positive controls and polymyxin-B solution in phosphate buffer solution as negative control. Isolates of *E. coli* recovered from SMAC plates and Stx-positive were O and H typed with commercial O157 and H7 sera (Difco Detroit, MI, USA). Isolates of STEC were also serogrouped with 12 commerical EPEC antisera.

### Serogrouping of EPEC

Strains biochemically identified as *E. coli* were subjected to slide agglutination test using polyvalent and appropriate monovalent EPEC O-specific antisera. All clinical strains were serogrouped and classified into different serogroups of EPEC (O26, O55, O86, O111, O114, O119, O124, O125, O126, O127, O128, and O142) using O-specific antisera according to the instructions of the manufacturer (Bio-Rad Co). A strain giving clumping with 4% saline was defined as rough.

### Statistical analysis

The differences among groups were analyzed by the chi-square and the Fisher's exact test. Chi-square was used for observed frequency more than five and the Fisher's exact test for observed frequency less than five. P values less than 0.05 were considered significant for all tests.

## RESULTS

### Study population

In total, 1,320 children were randomly selected, of whose 28 refused to provide faecal samples, Finally, 672 samples from Mazandaran province and 620 samples from Ilam province were studied, i.e. 1,292 children—612 (47.4%) females and 680 (52.6%) males—were included in the study. Their mean age was 5.2 years (range 6 months to 10 years).

### Prevalence of diarrhoea according to sex and age

In Ilam and Mazandaran provinces, diarrhoea was prevalent among 96 (14.8%) and 88 (13%) children respectively. No significant correlation was found between diarrhoea and sex. The prevalence of diarrhoea was highest (30%) among children aged less than two years.

### Prevalence of bacterial pathogens in diarrhoeal and asymptomatic persons

Of the 1,292 children studied, 184 were suffering from diarrhoea, and the remaining had no diarrhoea. Table 1 shows the rates of isolation of different bacterial pathogens from children with and without diarrhoea. *Campylobacter* spp. was not sought in this study. No bacterial pathogens could be isolated from the remaining 85 persons with diarrhoea.

**Table 1.** Isolation of pathogens from stools of children with and without diarrhoea

Pathogen	Criteria					
	With diarrhoea No. (%)			Without diarrhoea No. (%)		
	Ilam (n=96)	Mazandaran (n=88)	Total (n=184)	Ilam (n=524)	Mazandaran (n=584)	Total (n=1,108)
EPEC	39 (40.6)	28 (31.8)	67 (36.4)	44 (8.4)	36 (6.2)	80 (7.2)
STEC	12 (12.5)	4 (4.5)	16 (8.7)	21 (4)	2 (0.4)	23 (2)
*Shigella spp.*	6 (6.2)	3 (3.4)	9 (4.9)	1 (0.2)	0 (0.0)	1 (0.1)
*Salmonella spp.*	2 (2)	4 (4.5)	6 (3.2)	0 (0.0)	0 (0.0)	0 (0.0)
*Vibrio cholerae*	0 (0.0)	0 (0.0)	0 (0.0)	0 (0.0)	0 (0.0)	0 (0.0)
*Aeromonas spp.*	0 (0.0)	1 (1.1)	1 (0.5)	2 (0.4)	1 (0.2)	3 (0.3)
*Yersinia spp.*	0 (0.0)	0 (0.0)	0 (0.0)	0 (0.0)	0 (0.0)	0 (0.0)

EPEC=Enteropathogenic *Escherichia coli*;

STEC=Shiga toxin-producing *Escherichia coli*

### Prevalence of EPEC and STEC among diarrhoeal and asymptomatic persons

EPEC was isolated from 67 (36.4%) of the 184 children with diarrhoea compared to 80 (7.2%) of the 1,108 children without diarrhoea (p<0.05). The prevalence of EPEC was highest among children with diarrhoea. Eight (4.4%) of the 184 children with diarrhoea were colonized with two different serogroups of EPEC, while no asymptomatic children were colonized with more than one serogroup of EPEC. Table 2 shows the comparison of isolation rates of EPEC serogroups in patients and controls. The O111, O127 and O142 serogroups had the highest prevalence. Thirty-three (5.3%) faecal samples from Ilam and six (0.9%) from Mazandaran were STEC-positive (Table 1). In total, 16 (8.7%) samples from the diarrhoeal cases and 23 (2%) samples from the asymptomatic cases were positive for STEC strains. A significant association (p<0.05) was found between STEC and diarrhoeal illness. One strain of STEC isolated in the study belonged to the O26 serogroup and was isolated from the diarrhoeal cases. However, the O157:H7 serotype and mixed infections were not observed. None of the non-sorbitol fermentation strains agglutinated with O157 antisera.

**Table 2.** Serogroups of EPEC isolated from children with and without diarrhoea

Serogroup	No. (%) of infected children															
	With diarrhoea (n=184)								Without diarrhoea (n=1,108)							
	Age-group (years)								Age-group (years)							
	<2 (n=54)		2–5 (n=68)		6–10 (n=62)		Total (n=184)		<2 (n=83)			2–5 (n=393)		6–10 (n=632)		Total (n=1,108)	
	No.	%	No.	%	No.	%	No.	%	No.	%	No.	%	No.	%	No.	%
0111	7	36.8	7	36.8	5	26.4	19	10.3	3	9.3	18	56.2	11	34.4	32	2.9
0127	7	50	5	35.7	2	14.3	14	7.6	2	14.3	7	50	5	35.7	14	1.3
0142	5	41.7	4	33.3	3	25	12	6.5	3	30	2	20	5	50	10	0.9
0126	4	57	1	14.3	2	28.6	7	3.8	1	33.3	1	33.3	1	33.3	3	0.3
0119	2	66.7	0	0.0	1	33.3	3	1.6	2	66.7	1	33.3	0	0.0	3	0.3
0128	1	33.3	2	66.7	0	0.0	3	1.6	1	33.3	2	66.7	0	0.0	3	0.3
026	1	50	0	0.0	1	50	2	1.1	0	0.0	2	100	0	0.0	2	0.2
055	2	100	0	0.0	0	0.0	2	1.1	0	0.0	3	75	1	25	4	0.4
086	0	0.0	2	100	0	0.0	2	1.1	0	0.0	2	33.3	4	66.7	6	0.5
0125	1	50	0	0.0	1	50	2	1.1	0	0.0	1	100	0	0.0	1	0.09
0114	0	0.0	1	100	0	0.0	1	0.5	0	0.0	1	50	1	50	2	0.2
Total	30	16.3	22	11.9	15	8.2	67	36.4	12	1.1	40	3.6	28	2.5	80	7.2

## DISCUSSION

Acute infectious diarrhoea is a ubiquitous prevailing disease everywhere in the world. Its annual incidence in Asia, Africa, and Latin America is much higher than that in the rest of the world (1). In Iran, the disease is very common, particularly in the summer season when the number of patients referring to hospitals and clinics is unimaginably high (3).

In the present study, strains of EPEC were identified in 36.4% of the diarrhoea cases and 7.2% of children without diarrhoea (p<0.05). The results of our study showed that EPEC strains were an important cause of acute gastroenteritis, particularity in infants. In some studies, EPEC has been found to be a cause of 60–80% diarrhoea in children (21–22). Two studies in Iran have reported the prevalence of EPEC in diarrhoeal disease. One study in Tehran, capital of Iran, revealed that 9.4% of 502 cases harboured EPEC (3), belonging to a different serogroup, of which O126 and O55 were the most common ones. Results of another study conducted in southern Iran showed that 31% of cases of acute diarrhoea were due to serogroups of EPEC, and serogroups O128 and O126 were more frequently found (23).

The rates of isolation of enteric pathogens reported in different studies are related to socioeconomic, health, and weather conditions. EPEC is commonly transmitted via the faecal-oral route in a poor hygienic environment (24). Results of studies carried out in different areas with different health and socioeconomic status showed that strains of EPEC were the major cause of diarrhoea in developing countries (3, 23).

Pure isolates of *E. coli* are frequently obtained from diarrhoeal stools of children. However, since serotyping facilities are not available in all routine laboratories around the world, it is difficult to determine whether diarrhoea was due to EPEC and it can be considered to have a significant causal role or is a member of normal commensal flora.

In the past, the most prevalent serogroups of EPEC in Iran were O126, O55, and O128 (3, 23), whereas O111 was dominant, particularly between the two populations we have studied. Results of these studies showed that EPEC continues to be associated with endemic infantile diarrhoea in Iran. Furthermore, these bacteria are the most frequent agents of diarrhoea in children. The association of certain serogroups of EPEC with endemic infantile diarrhoea has also been reported from several countries, including Bangladesh (6), Saudi Arabia (11), Brazil (25), Uruguay (2), and in several studies (3, 23). Results of a study in Iran showed that 65% of isolates identified by commercial antisera were probably a putative EPEC (26), and although O serogrouping is not a perfect tool, in our setting it is still a useful method for presumptive identification of these strains.

In the present study, 12.5% and 4.5% of diarrhoeal cases were infected with STEC isolates in Ilam and Mazandaran province respectively, while healthy children with STEC in these provinces were only 4% and 0.4% respectively (Table 1). There was a significant association between STEC-associated infection and diarrhoea. In previous studies, rates of isolation of STEC strains in diarrhoeal cases within the age range of 1–80 year(s) were 16.7% and 0.6% in the summer and spring seasons respectively (13, 14). None of the STEC strains belonged to the O157:H7 serotype. These data reconfirmed the geographical variation and showed the absence of the O157:H7 serotype among STEC isolates in Iran. There may be many risk factors for young children, such as inadequate personal hygiene, childhood habits, like nail-biting and thumb-sucking, and close contact with domestic animals (27).

Several studies have reported that O157:H7 is an important cause of diarrhoea, either as the sole causative agent or part of STEC isolates, with an incidence rate close to those of *Salmonella* and *Campylobacter* (28–30), whereas data concerning non-O157 strains are rare. This probably is due to the fact that most commercially-available diagnostic tests are serotype-specific and are also expensive for routine detection in clinical laboratories (31).

Isolation of STEC among non-diarrhoeal cases could be attributed to their traditional life-style in rural areas. Close proximity with their livestock and drinking of unpasteurized milk would give rise to immunity against Shiga toxin over the lifespan (32).

In conclusion, the results of our study revealed that the high frequency of various serogroups of EPEC among young children is a major public-health problem. A significant association between isolation of STEC and diarrhoea was observed. Further epidemiological studies are needed, and these should include prevalence of various EPEC serotypes and other diarrhoeagenic *E. coli.*

## ACKNOWLEDGEMENTS

The authors are grateful to Dr. Y. Takeda for providing them with standard strains and anti-stx-1/stx-2 antisera, to Dr. S. Bouzari and Dr. H. Shojaei for their critical discussion, and to Mr. J. Reasi, Mr. H. Khamseh, and Mrs. M. Lotfi for their technical help. The authors also thank the authority and personnel of the health department of Ilam and Mazandaran provinces.
